# Stratification of *Archaea* in the Deep Sediments of a Freshwater Meromictic Lake: Vertical Shift from Methanogenic to Uncultured Archaeal Lineages

**DOI:** 10.1371/journal.pone.0043346

**Published:** 2012-08-21

**Authors:** Guillaume Borrel, Anne-Catherine Lehours, Olivier Crouzet, Didier Jézéquel, Karl Rockne, Amélie Kulczak, Emilie Duffaud, Keith Joblin, Gérard Fonty

**Affiliations:** 1 Laboratoire « Microorganismes : Génome et Environnement », UMR CNRS 6023, Clermont Université (Université Blaise Pascal and Université d’Auvergne), Aubière, France; 2 Laboratoire de Géochimie des Eaux, Institut de Physique du Globe de Paris, UMR CNRS 7154, Université Paris 7, Paris, France; 3 Department of Civil and Materials Engineering, University of Illinois at Chicago, Chicago, Illinois, United States of America; University of Waterloo, Canada

## Abstract

As for lineages of known methanogens, several lineages of uncultured archaea were recurrently retrieved in freshwater sediments. However, knowledge is missing about how these lineages might be affected and structured according to depth. In the present study, the vertical changes of archaeal communities were characterized in the deep sediment of the freshwater meromictic Lake Pavin. For that purpose, an integrated molecular approach was performed to gain information on the structure, composition, abundance and vertical stratification of archaeal communities thriving in anoxic freshwater sediments along a gradient of sediments encompassing 130 years of sedimentation. Huge changes occurred in the structure and composition of archaeal assemblages along the sediment core. Methanogenic taxa (*i.e. Methanosaeta* and *Methanomicrobiales*) were progressively replaced by uncultured archaeal lineages (*i.e.* Marine Benthic Group-D (MBG-D) and Miscellaneous Crenarchaeal Group (MCG)) which are suspected to be involved in the methane cycle.

## Introduction

The high densities of viable prokaryotes in marine or freshwater sediments (between 10^9^ and 10^10^ cells.cm^−3^ of sediment) have prompted microbiologists to consider the metabolic roles of the sediment microbiota in the cycling of nutrient elements [1]: oxidation of deposited organic matter, regeneration of inorganic nutrients and transformation of those inorganic materials [2]. In those environments, the complete mineralization of complex organic matter involves a variety of anaerobic prokaryotes operating in close interactions [3]. These microbial interactions are a classic example of microbial interdependency following the concept of the “anaerobic food chain” borrowed from ruminant microbiology [4]. In anaerobic sediments, these processes may be viewed as sequential in space or in time, as recently deposited material moves deeper into the sediments and through different “microbial zones” successively dominated by a terminal-electron-accepting process (i.e. denitrification, iron reduction, sulfate-reduction and methanogenesis [5]).

**Figure 1:**
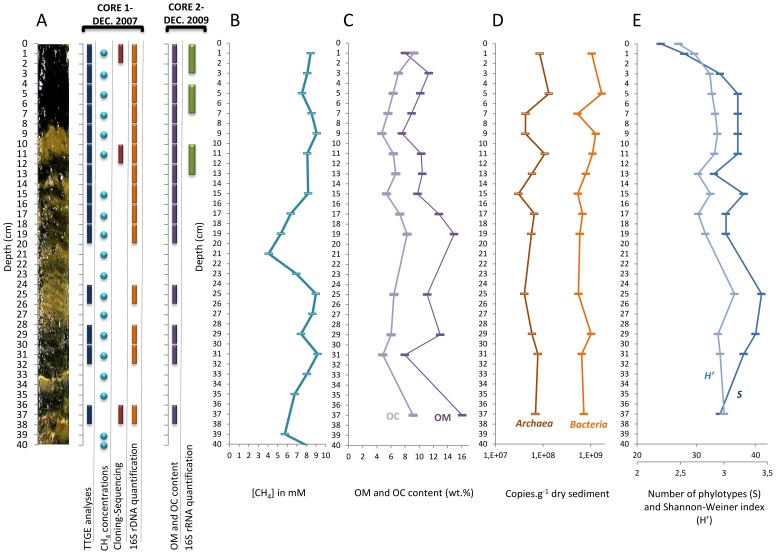
Sampling strategy and stratification of abiotic and biotic features in Lake Pavin sediments. (A) Sampling strategy: the depths where analyses were performed on each sediment core are indicated by circles or rectangles. (B) Methane profile (concentration in mM) along the sediment core 1. (C) Organic matter (OM) and organic carbon (OC) content determined from Lake Pavin sediment core 2. (D) Abundance of *Bacteria* and *Archaea* along the sediment core 1, each symbol represents the average value of duplicate quantifications. (E) Vertical changes of the number of archaeal phylotypes (richness, S, on the horizontal axis 20 to 40) and diversity (Shannon diversity index, H’, on the horizontal axis 2 to 3.5) along the sediment core 1. The number of phylotypes corresponds to the number of bands on the TTGE gel. The diversity was calculated according to the number of TTGE bands and their relative intensity.

Whereas these global patterns remain valid, research performed in the last two decades has extended our knowledge of the diversity and function of the sedimentary microbiota, particularly those of the “most enigmatic of life’s three domains”, the *Archaea*. A striking example is the discovery of the anaerobic methane oxidation process (AOM) performed by a microbial consortium involving the ANaerobic MEthanotrophs (ANME) archaea in marine sediments [6]–[8], and probably other archaeal groups in freshwater systems [9]. Another major advance is the identification of number of uncultured archaeal lineages with unknown functions in ecosystems with moderate environmental conditions, including marine and freshwater sediments [10].

In comparison to marine sediments, archaeal community structure and composition were less documented in freshwater lake sediments and studies were mainly focused on the methanogenic archaea of the surficial sediment layer [11]–[14]. While a general view of the dominant archaeal lineages inhabiting freshwater sediments has begun to emerge from these studies, knowledge is missing about how these lineages might be affected and structured according to depth and changing environmental factors. Among freshwater systems, meromictic lakes are rare and of considerable interest to microbial ecologists because their permanent anoxic layers exhibit potential undisturbed climax microbial communities and because of their relationship to an earlier biosphere [15]. Lake Pavin in France provides such an environment. The sedimentary compartment of Lake Pavin, permanently surmounted by a 30 m anoxic water column, is thus unusual and provides a special opportunity for investigation of the sediment microbiota, *e.g*. stability of physical parameters (temperature, sedimentation rates), steady state of the above anoxic water column and low availability of inorganic electron acceptors [16]–[19].

The present study is an exploratory work to investigate the structure and composition of archaeal communities thriving in anoxic freshwater sediments along a gradient of sediments encompassing 130 years of sedimentation. The issues raised in this study are as follows: (i) is archaeal communities important component (quantitative) of microbial communities inhabiting this environment, (ii) are methanogenic groups dominating the *Archaea*? and if not, which archaeal groups are retrieved? (iii) is Archaea exhibiting a spatial pattern consistent with changes in environmental factors (*e.g.* organic matter). To these aims, an integrated fine-scale microbial community structure analysis was performed, using multiple molecular approaches including fingerprint patterns, quantitative polymerase chain reaction (qPCR) and 16S rRNA analyses.

**Table 1:** Primers and amplification features used in this study.

Approach	Targeted group	Primer set	Sequence (5′ –>3′)	Amplicon size	Annealing T°C	Reference
**Cloning**	*Archaea*	21f	TTCCGGTTGATCCYGCCGGA	1360	60	[25]
		1386r	GCGGTGTGTGCAAGGAGC			[26]
**TTGE**	*Archaea*	934fGC*	GAATTGGCGGGGGAGCAC	490	60	[26]
		1386r	GCGGTGTGTGCAAGGAGC			[26]
**qPCR**	*Bacteria*	BAC338f	ACTCCTACGGGAGGCAG	470	60	[41]
		515r	ATTACCGCGGCTGCTGGCA			[39]
	*Archaea*	934f	GAATTGGCGGGGGAGCAC	105	60	[26]
		1040r	GGCCATGCACCWCCTCTC			[40]
	*Crenarchaeota*	771f	ACGGTGAGGGATGAAAGCT	227	60	[38]
		957r (modified)	CGGCGTTGACTCCAATT**R**			*This study* **
	*Methanomicrobiales*	MMB282f	ATCGRTACGGGTTGTGGG	506	60	[41]
		MMB832r	CACCTAACGCRCATHGTTTAC			[41]
	*Methanosaetaceae*	Mst702f	TAATCCTYGARGGACCACCA	164	60	[41]
		Mst862r	CCTACGGCACCRACMAC			[41]
	MBG-D	490f	GAGAGTAAGRGCTGGGTA	348	58	*This study*
		818r	ACTAACATCAAGCRAGCAG			*This study*

* GC clamp at the 5-end: CGC CCG CCG CGC GGC GGG CGG GGC GGG GGC ACG GGG [32].

** the “R” in bold replace a “G” in the original primer designed by [38]. MBG-D: Marine Benthic Group-D.

## Materials and Methods

### Site Description

*No specific permits were required for the described field studies, as the location is not privately-owned or protected in any way, and the field studies did not involve endangered or protected species.*

Lake Pavin, located at 45°55 N and 2°54 E, is the youngest volcano crater lake in the French Massif Central (6,000 years BP). Lake Pavin has a circular shape, an area of 0.44 km^2^ and a maximum depth of 92 m, at an elevation of 1,197 m above sea level. Lake Pavin is the unique meromictic lake in France and is characterized by the presence of two permanent stratified layers. The upper layer (mixolimnion) extends from the surface to 60 m depth and is affected by mixing during fall and spring. The deepest layer (monimolimnion) extends from 60 to 92 m depth and includes the chemocline (60- to 70-m). The conductivity in the monimolimnion increases from 40 to 340 µS.cm^−1^ [17] and is in the range of that measured in freshwater lakes. Depth profiles of nutrients, oxygen, temperature, conductivity, methane, carbon dioxide, sulfate, nitrate, iron, and trace element concentrations in the anoxic water column have been published previously [16]–[20]. Two papers were also related to bacterial counts, structure and phylogenetic diversity of bacterial and archaeal communities in the anoxic zone of Lake Pavin [15], [20].

### Sampling Procedure

Two 40 cm-sediment cores, representing approximately 130 years of sedimentation history of the lake [21], were sampled in December 2007 (sediment core 1) and in December 2009 (sediment core 2), at the maximum depth zone of the lake, using an Uwitech gravity corer. The sediment cores were sectioned into segments and homogenized aseptically under N_2_ flux to preserve anaerobic conditions. Sub-samples of the homogenized sections were stored at −20°C for DNA-based analyses. On sediment core 2, subsamples were pooled and were frozen in the field and kept at −80°C until RNA extraction. The sampling strategy and the experiments conducted on the different sediment-cores are presented in the Figure 1A. Although no temporal study has been conducted on Lake Pavin sediments, the steady state of the anoxic water column [16]–[19] suggests that temporal variations in the physicochemical composition of the sedimentary compartment are limited. Accordingly, temporal changes in the stratification of benthic microbial communities are expected to be low. This assumption is supported by a study on the meromictic Lake Kaiike which has reported minor seasonal changes in the stratification of benthic bacterial communities [22]. Physicochemical and biological differences between the two sediment cores analyzed in the present study are obviously not excluded. However, in the particular context of meromictic lakes and considering that sampling was performed in the same season (December), data from the two sediment cores were interpolated.

**Figure 2:**
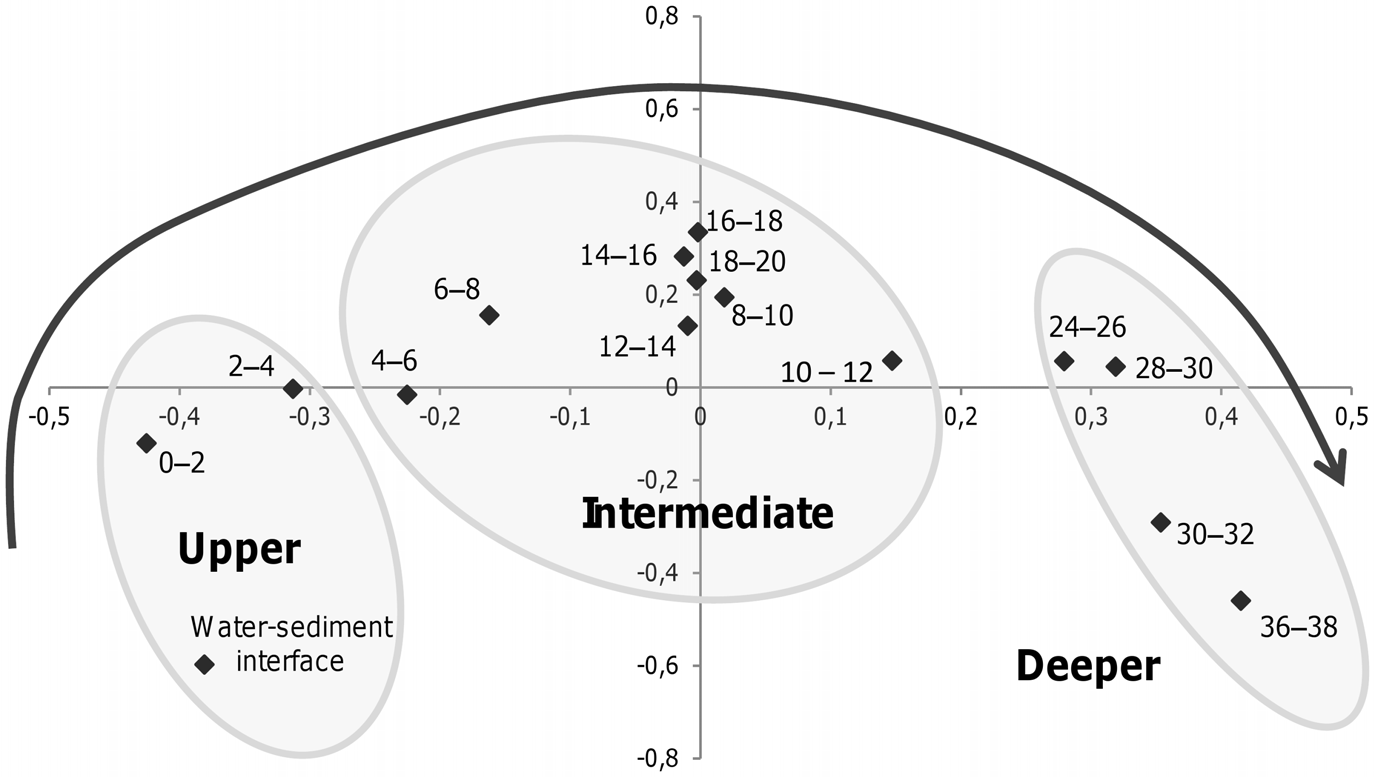
Multidimensional scaling plot (MDS) of the archaeal community based on TTGE profiling of archaeal 16S rRNA genes. This plot corresponds to a two-dimensional visualization of the Jaccard distance matrix. The ellipses designate clusters exhibiting more than 55% similarity and delineate the upper layer (0–3 cm depth), the intermediate layer (4–20 cm depth) and the deeper layer (24–38 cm depth) of the sediment core 1.

**Figure 3:**
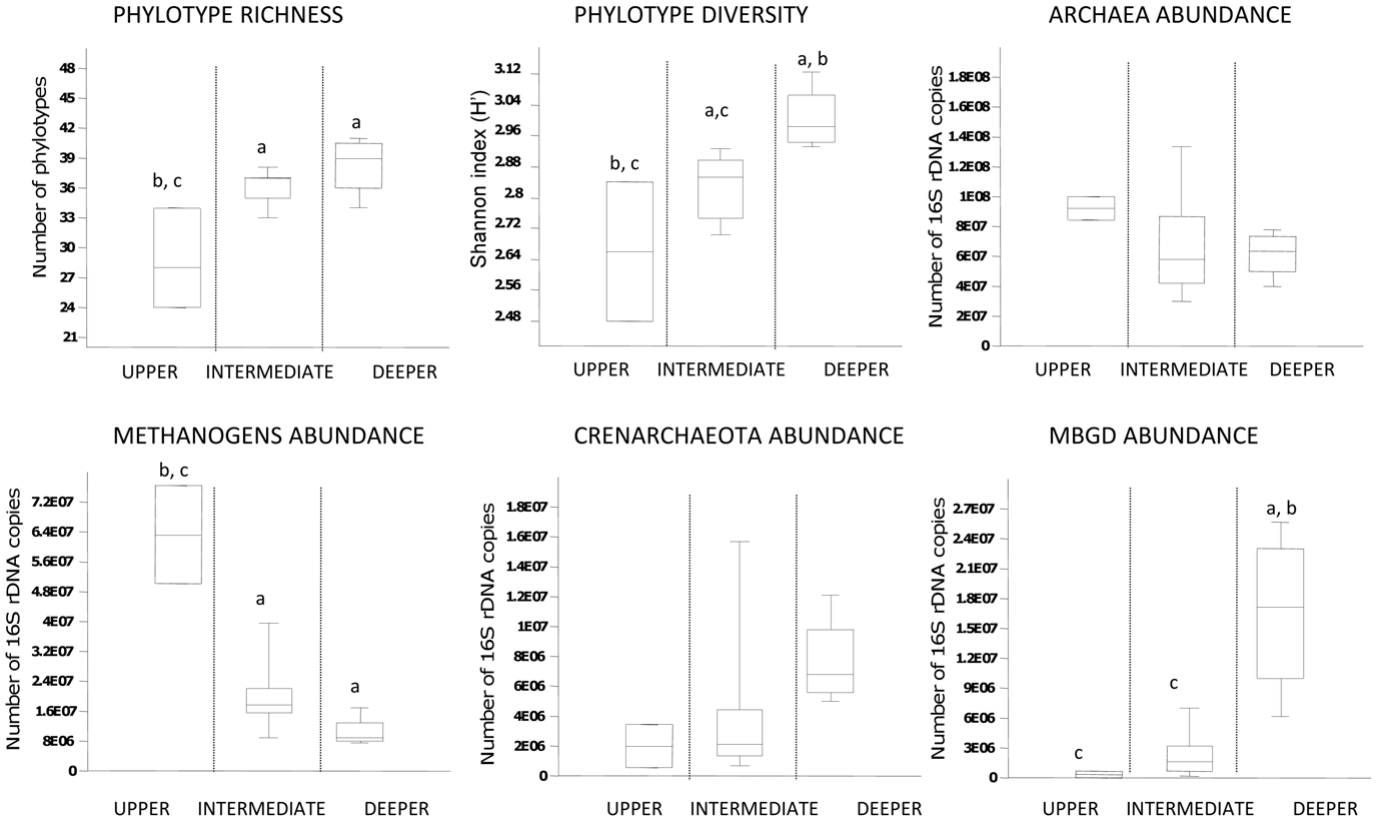
Box plots of richness, diversity and abundance for the *Archaea,* methanogens, *Crenarchaeota* and MBG-D. The three layers were discriminated from TTGE profile analyses. a, b, c indicate significant differences (p<0.05). Richness and diversity were calculated from the number and the relative peak area of bands on TTGE profiles. Average abundances were determined from 16S rDNA q-PCR data. The abundance of the methanogens was estimated by addition of the abundance of *Methanosaetaceae* and *Methanomicrobiales*. All the data were obtained on sediment core 1.

**Figure 4:**
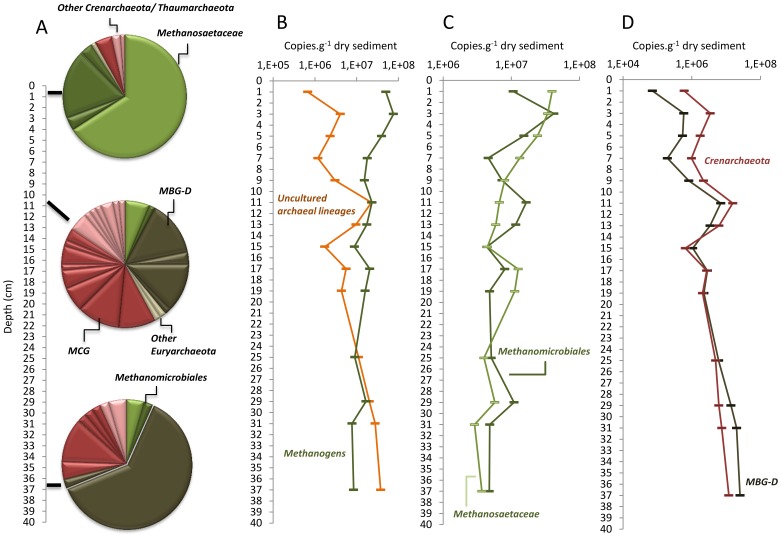
Frequency and abundance of the archaeal lineages. (A) Frequency of archaeal lineages in clone libraries constructed from samples collected at 0–2 cm, 10–12 cm and 36–38 cm in the sediment core 1. Segments in the circles correspond to OTUs grouping sequences with >97% similarity. (B) Abundance of the methanogenic (*Methanosaetaceae*+*Methanomicrobiales*) and uncultured archaeal lineages (MBG-D+*Crenarchaeota*). (C) Abundance of the *Methanomicrobiales* and *Methanosaetaceae*; (D) Abundance of the MBG-D and *Crenarchaeota*. Each symbol represents the average value of the duplicate of 16S rDNA quantification from samples collected in the sediment core 1. MBG-D: Marine Benthic Group-D, MCG: Miscellaneous Crenarchaeotal Group.

**Figure 5:**
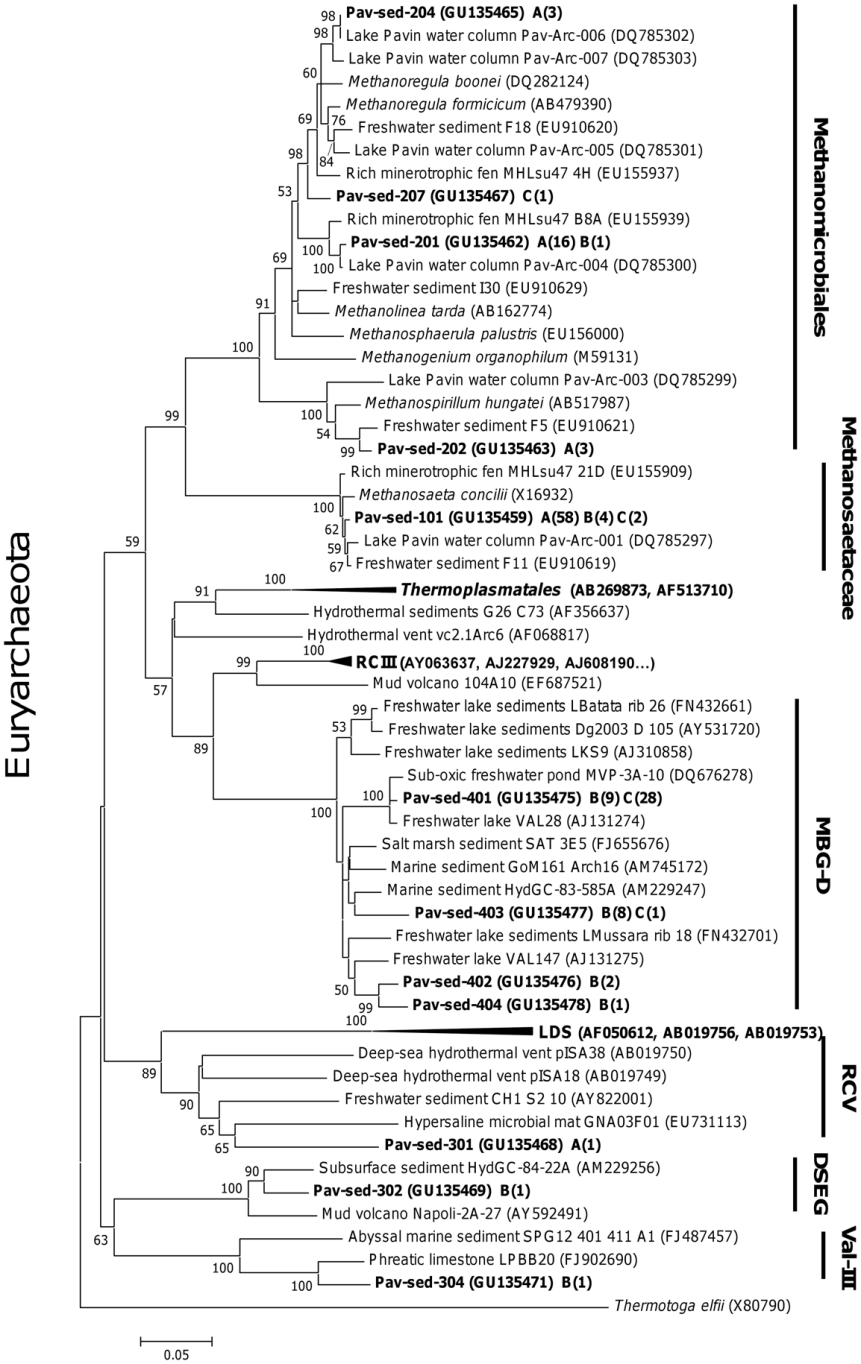
Neighbor-joining tree of the euryarchaeotal 16S rRNA genes. Bootstrap values (in percent) are based on 1000 replicates and are indicated at nodes for branches values ≥50% bootstrap support. The number of sequences within each OTU is shown in parentheses, and the depths of samples used for clone library construction is indicated by a letter: A, B, C for 0–2 cm, 10–12 cm and 36–38 cm depth, respectively. The scale bar represents a 5% sequence difference, and *Thermotoga elfii* was used as an out-group. Sequences were obtained from sediment core 1. MBG-D: Marine Benthic Group D, LDS: Lake Dagow Sediments, RCIII: Rice Cluster III, RCV: Rice Cluster V, DSEG: Deep Sediment Euryarchaeotal Group, Val-III: Valkea-III.

**Figure 6:**
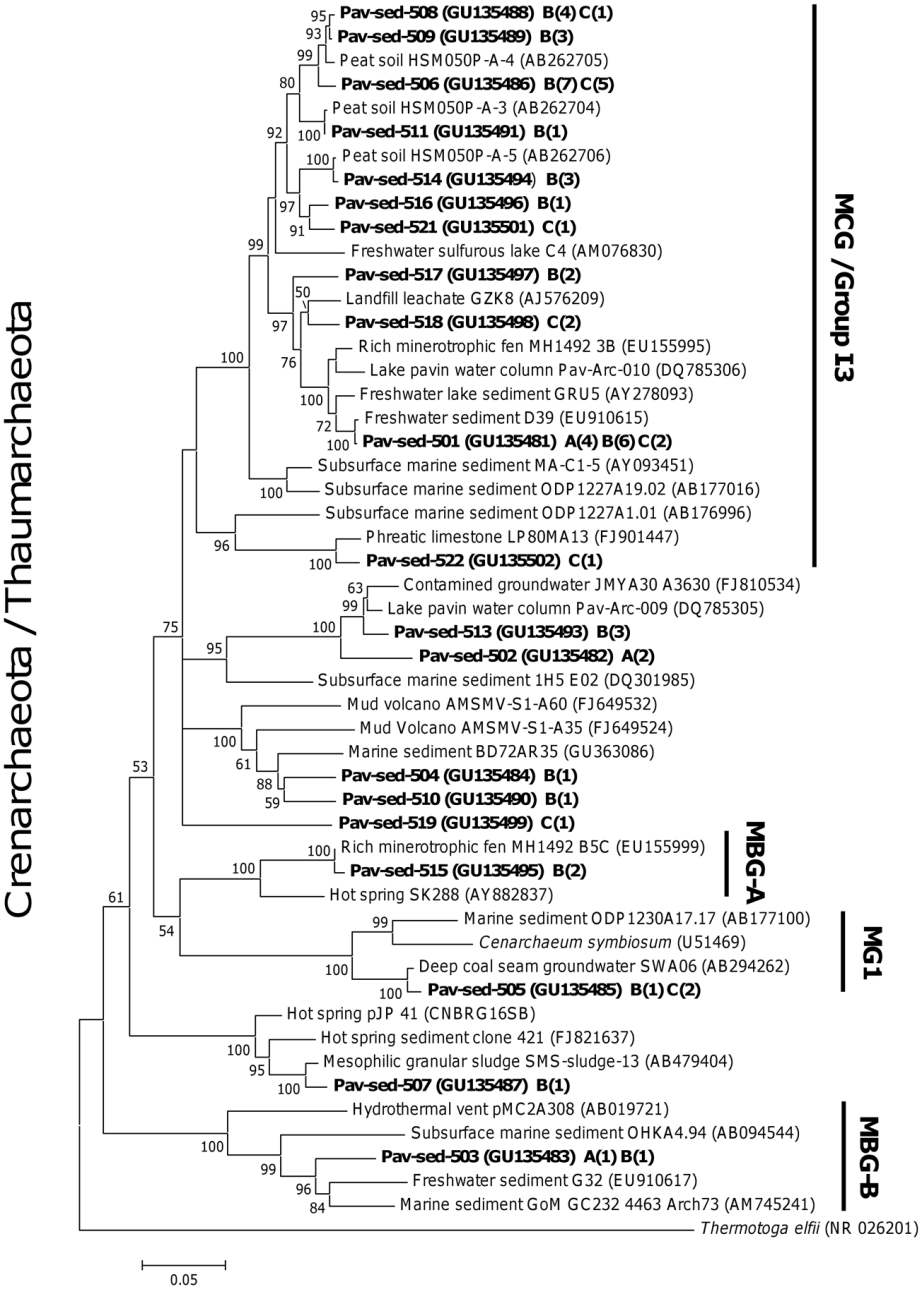
Neighbor-joining tree of the crenarchaeotal/thaumarchaeotal 16S rRNA genes. Bootstrap values (in percent) are based on 1000 replicates and are indicated at nodes for branches values ≥50% bootstrap support. The number of sequences within each OTU is shown in parentheses, and the depths of samples used for clone library construction is indicated by a letter: A, B, C for 0–2 cm, 10–12 cm and 36–38 cm depth, respectively. Data were obtained from sediment core 1. The scale bar represents a 5% sequence difference, and *Thermotoga elfii* was used as an out-group. Sequences were obtained from sediment core 1. MCG: Miscellaneous Crenarchaeotal Group, MBG-A: Marine Benthic Group A, MBG-B: Marine Benthic Group B, MGI: Marine Group I.

### Organic Carbon and Methane Analyses

Organic carbon and organic matter were measured using a modification of the procedure outlined in Buckley *et al*. [23]. Subsample of homogenized sediment (3 mL) was dried to constant mass for analysis of bulk density, water content, and porosity using standard methods. Dried sediment aliquots were placed in an acid fumer for 24 h to remove inorganic carbon and subsequently dried. These samples were split for organic carbon analysis using a Carlo Erbo elemental analyzer, or combusted at 375°C for 24 h in air for the oxidation of heat-labile organic matter components. The latter fraction is operationally termed “organic matter” as described previously [23]. Methane concentrations were determined as described by Lopes *et al.* [17].

### DNA and RNA Extraction

Genomic DNA was extracted using the Ultra clean® soil DNA extraction kit (MOBio Laboratories, Inc) according to the manufacturer’s instructions. Total RNA was extracted and separated from DNA as previously described by Purdy *et al.* [24]. Residual DNA was digested with Ambion® TURBO DNA-free kit (Applied Biosystems) according to rigorous DNase treatment protocol. According to this procedure the step of DNA digestion was repeated one more time and the volume of DNase inactivation reagent was doubled.

### Clone Library Construction and Phylogenetic Analyses

Archaeal 16S rRNA genes (hereafter referred to as “16S rDNA” for a better distinction with 16S rRNA transcripts) were amplified using the set of primers 21f and 1386r (Table 1, [25], [26]). The PCR reaction mixture (50 µl) contained 5 µl of 10 X reaction buffer, 2.5 mM MgCl_2_, 200 µl of each deoxyribonucleotide triphosphate (dATP, dCTP, dGTP, dTTP; Eurobio), 200 nM of each primer, 250 ng/µl of bovine serum albumin (BSA), 1.5 U of Taq Hotstar (Qiagen) and 1 µl of genomic DNA.

**Figure 7:**
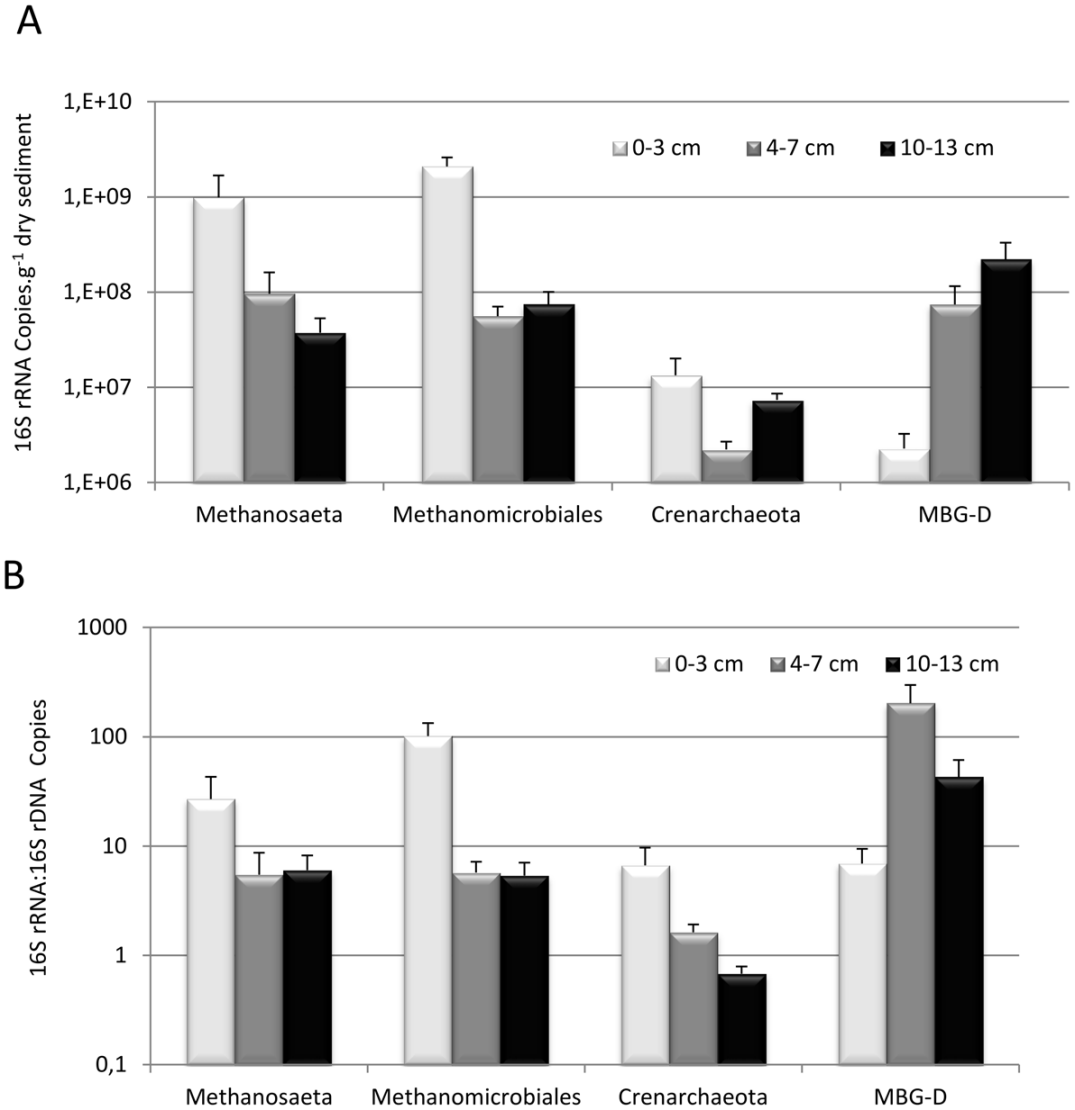
Activity of the main archaeal lineages. (A) Activity of the methanogens, *Crenarchaeota* and MBG-D was estimated by the reverse transcription and PCR quantification of their 16S rRNA; (B) 16S rRNA to 16S rDNA ratio of the methanogens, *Crenarchaeota* and MBG-D. Quantifications of 16S rRNA were performed on the sediment core 2, whereas those of 16S rDNA were performed on sediment core 1. MBG-D: Marine Benthic Group-D.

Amplifications were performed with a PTC-200 thermal cycler (MJ Research) using the following program: a 15 min hot start at 95°C, followed by 35 cycles consisting of denaturation (1 min at 95°C), annealing (1 min at 58°C) and extension (1 min at 72°C), with a final extension for 10 min at 72°C. PCR products were cloned using a TOPO TA cloning Kit according to the manufacturer’s instructions (Invitrogen). Cloned inserts were PCR amplified using the M13 forward and reverse primers and amplicons were digested with the restriction endonuclease HaeIII (Qbiogene) at 37°C for 12 h. Digests were analyzed by gel electrophoresis using 2.5% (wt/vol) Nusieve 3∶1 agarose (Tebu-Bio) gels containing ethidium bromide (0.5 mg.L^−1^) normalized with a 100 bp size marker (Invitrogen). Visual analyses of restriction fragment length polymorphism (RFLP) patterns were performed and banding patterns were grouped according to similarity. Plasmid DNAs from representatives of each RFLP pattern were isolated using the QIAprep plasmid purification kit (Qiagen, Chatsworth, Calif.). Clones were sequenced by Eurofins MWG Operon (Ebersberg, Germany) using M13 forward and reverse primers, and clone libraries were screened for chimeric sequences with the Bellerophon program available at http://foo.maths.uq.edu.au~/huber/bellerophon.pl [27].

Sequences were compared to available databases using the BLAST network service to determine approximate phylogenetic affiliations. Sequences were aligned with CLUSTAL W [28], and phylogenetic trees were computed using neighbor-joining approaches with MEGA 5 software (available at http://www.megasoftware.net/ [29]). The robustness of inferred topologies was tested by bootstrap analysis and 1000 resamplings of trees. Sequences exhibiting more than 97% of similarity were grouped into the same Operational Taxonomic Unit (OTU). Coverage values of clone libraries were calculated as previously described [30]. Sequences were deposited in Genbank under accession No. GU135459-GU135502. Sorensen similarity (Cs) and Bray Curtis dissimilarity indices were calculated as previously described [31].

### Temporal Temperature gel Gradient Electrophoresis (TTGE) Analyses

Archaeal 16S rDNA were amplified using the set of primers 934f-GC/1386r (Table 1, [26], [32]). The reaction mixture (50 µl) contained the same component as described above (see clone library construction). A touch-down PCR was performed using the following program: a 15 min hot start at 95°C, 5 cycles consisting of denaturation (1 min at 95°C), annealing (1 min at 65°C) with a decrease of 1°C per cycle, and extension (1 min at 72°C), followed by 30 cycles consisting of denaturation (1 min at 95°C), annealing (1 min at 60°C), and extension (1 min at 72°C), with a final extension for 10 min at 72°C. The PCR products were quantified with the DNA quantitation kit fluorescence assay (Sigma-Aldrich) and 300 ng of each sample were electrophoresed through a 8% polyacrylamide gel (TAE 1.25 x, urea 7 M, Temed 0.06%, ammonium persulfate 0.0625%) as previously described [33]. Band patterns were analyzed using the GelCompare 4.6 software package (Applied Maths, Kortrijk, Belgium). A 1% band position tolerance (relative to total length of the gel) was applied in band assignment, which indicates the maximal shift allowed for two bands in different TTGE tracks to be considered as identical. Pairwise similarity matrices were calculated using the Jaccard equation from presence/absence data. Dendrograms were generated using UPGMA method [34]. A distance of 40% was used to separate clusters in the hierarchical classification. Relationships among samples were visualized using the ordination technique multidimensional scaling (MDS) using a standardized stress with 1000 iterations computed with XLSTAT version 6.01. Analysis of similarity (ANOSIM, [35]) was used to test the hypothesis that communities within each cluster were more similar to each other than to communities in other clusters. The number of bands in a profile was expressed as the phylotype richness (S). The Shannon diversity index (H’) was calculated from the number of bands and their relative intensity.

### Quantitative PCR and RT-qPCR Analyses

#### PCR primer design and modification

The primers 490f and 818r targeting MBG-D were designed in this study (Table 1, Figure S1) and were analyzed with Beacon Designer program (available at http://www.premierbiosoft.com/qOligo/Oligo.jsp?PID=1) in order to avoid hairpins, self- and heterodimers. Specificity of primers 490f and 818r was checked *in silico* using ProbeCheck ([36], available at http://www.microbial-ecology.net/probecheck) querying RDP II database version 9.61 [37]. *In situ* analysis, performed by sequencing clone libraries constructed with the set of primers 490f and 818r (according to experimental procedure described above), confirmed primer specificity. The primer 957r targeting *Crenarchaeota* [38] was modified (R instead of G at the 3′ end) to remove one mismatch with most of the *Crenarchaeota* sequences retrieved in clone libraries constructed in this study (Table 1). With this modification, the sequences of the main euryarchaeotal lineages found in Lake Pavin kept, at least, 4 mismatches with the two primers targeting the *Crenarchaeota*. In order to estimate the consequences of this modification, qPCR were performed with both modified and unmodified 957r primers on serial dilutions of plasmids containing 1380-bp partial 16S rDNA sequences of *Methanosaetaceae*, *Methanomicrobiales* and MBG-D. Aspecific amplifications were above the detection limit of the method only when concentrations of *Methanosaeta*, *Methanomicrobiales* and MBG-D were at least one order of magnitude above those detected in Lake Pavin samples.

#### 16S rDNA qPCR

*Archaea*, *Bacteria*, *Crenarchaeota*, *Methanosaetaceae*, *Methanomicrobiales* and MBG-D were quantified by real-time PCR using a specific set of primers (Table 1, [26], [38]–[41]). Real-time quantitative PCR analysis was conducted with a Mastercycler ep *realplex* (Eppendorf) using MESA green qPCR Master mix Plus for SYBR assay (Eurogenetec). In each qPCR run, besides community DNA and negative controls, a recombinant plasmid, linearized with the endonuclease XbaI, containing the 1380-bp partial 16S rDNA sequence was quantified using Quant-iT™ PicoGreen® dsDNA (Invitrogen) and was 10-fold serially diluted in triplicates ranging from 10^2^ to 10^9^ genome equivalents and used as templates to determine the standard curves by plotting the threshold cycle (CT) value against the logarithm of copy numbers (log Co) of 16S rDNA in each dilution. Amplification reactions contained the following: 12.5 µL of MESA green qPCR Master mix Plus for SYBR® assay (Eurogenetec), 400 mM of each primers, 250 ng.µL^−1^ of BSA, 2 µL of DNA template and water to a 25 µl final reaction volume. Runs were performed under the following conditions: a 5 min denaturation at 95°C, followed by 40 cycles of consisting of denaturation (30 s at 95°C), annealing (20 s at various temperatures (Table 1)), extension (30 s at 72°C), detection of SYBR green I signal measurement at (20 s at 80°C) and a final extension for 10 min at 72°C. Melting curve analysis was performed at the end of 40 cycles to ensure the proper amplification of targeted fragments and to investigate the differentiation of the 16S rDNA retrieved from the environmental samples. Fluorescence readings were consecutively collected during the melting process from 60 to 95°C. Fluorescence data were converted into melting peaks. All data were analyzed using Mastercycler ep *realplex* software (Eppendorf). CT values were used to determine the copy numbers of 16S rDNA in the environmental samples based on the standard curve. Quantifications were performed in duplicate. The number of 16S rDNA copies was converted into cell number assuming 2 and 3.8 copies of the 16S rDNA per archaeal and bacterial cell respectively [42].

#### 16S rRNA RT-qPCR

RNA extracts were diluted (1, 10 and 100 fold) and complementary DNA (cDNA) of archaeal 16S rRNA was obtained by using 1386r primer and the superscript III reverse transcriptase kit (Invitrogen) according to manufacturer’s instructions. Control amplifications with 21f and 1386r primers were carried out on samples treated with DNase before and after reverse transcription to check the purity of cDNA. Conditions for qPCR were similar as previously described for 16S rDNA (see above). Quantifications were performed in triplicate. The average 16S rDNA copy numbers from 0–4 cm, 4–8 cm and 10–14 cm in sediment core 1 were used to calculate the 16S rRNA to 16S rDNA copy number ratios.

### Statistical Analyses

Box plots and Spearman’s rank correlations were performed using Past software available at http://folk.uio.no/ohammer/past/ [43]. Significance was tested by one way-ANOVA.

## Results

### Archaeal Community Structure and Composition along the Sediment Core

*Archaea* exhibited gradual changes in community structure along the sediment core as profiled by TTGE of amplified 16S rDNA fragments (Figures 2 and S2). Groupings obtained from hierarchical cluster and from MDS analyses discriminated communities of the upper (0–4 cm depth) from those of the intermediate (4–20 cm depth) and of the deeper (24–38 cm depth) sediment layers (Figures 2 and S2A). These groupings were confirmed with ANOSIM statistics (Figure S2B). Both richness and diversity of the archaeal community increased with depth, particularly within the first centimeters (Figure 1E). Intermediate and deeper layers supported a significantly higher diversity of archaeal community than the surface layer (Figures 1E and 3). The large dissimilarity (>60%, Figure S2A) between clusters suggests that archaeal populations in deeper sediment layers clearly differed from those at the sediment surface. To confirm this observation and to identify archaeal taxa, the archaeal community composition was assessed by creating 16S rDNA clone libraries from samples at 0–2 cm, 10–12 cm and 36–38 cm depth of the sediment core (Figure 1A). A total of 199 clones were obtained and were grouped into different operational taxonomic units (OTU) according to their RFLP patterns. One representative clone of each OTU was sequenced and a total of 32 distinct archaeal sequences, according to a cut-off value of 97%, were subjected to phylogenetic analyses. The number of clones analyzed represented 91%, 64% and 74% coverage for clone libraries of the uppermost, intermediate and deep sediment layers, respectively. Sequences retrieved in this study were mainly affiliated to the two archaeal phyla, *Euryarchaeota* (∼ 70% of total sequences) and *Crenarchaeota* (∼ 30% of total sequences) (Figure 4A, Table S1), and one third of the sequences exhibited less than 95% of similarity with sequences available in public databases. The methanogenic lineages of *Methanomicrobiales* and *Methanosaetaceae*, and uncultured lineages Marine Benthic Group D (MBG-D) and Miscellaneous Crenarchaeal Group (MCG) accounted for 93% of archaeal clone sequences (Figure 4A, Table S1). Other lineages were detected but at low relative proportions (Figures 4A, 5 and 6): the RC-V [44], the Val-III [45], the Deep Sediment Euryarchaeotal Group (DSEG, [46], the Marine Benthic Groups A and B (MBG-A, MBG-B [47], the Marine Group-I (MGI [25]), and an unidentified lineage related to MCG. It should be noticed that the affiliation of several lineages detected in this study (MCG and MBG-B) within the “*Crenarchaeota*” is debated [48] as they could also be assigned to the base of the archaeal phylum “*Thaumarchaeota*” [49] or related to a more recently proposed archaeal phylum named “*Aigarchaeota*” [50]. Herein, they were referred as crenarchaeotal lineages. These lineages, and especially MCG, are phylogenetically diversified in Lake Pavin sediments and largely contributed to the high richness and diversity of the overall archaeal community in the intermediate and deeper layers of the sediment (Figures 1E and 6, Table S1).

The archaeal community composition exhibited a large heterogeneity with depth, with only 20% similarity between archaeal 16S rDNA sequences of 0–2 cm and 36–38 cm depths (Figure 4A). The uppermost layer was dominated by methanogenic lineages (66% of acetoclastic *Methanosaetaceae* and 25% of hydrogenotrophic *Methanomicrobiales*), whereas deeper in the sediment core, clone libraries were mainly composed of uncultivated archaeal lineages (62% of MBG-D and 26% of MCG, Figure 4A, Table S1). According to Bray-Curtis dissimilarity index, MBG-D and MCG lineages also exhibited different populations between samples of the intermediate and the deeper layers (33% and 57% of dissimilarity, respectively). Groupings of sediment depths with similar archaeal community structure, revealed by multivariate analysis of TTGE patterns, were then further supported by differences in phylogenetic composition of *Archaea*.

### 16S rDNA and 16S rRNA Quantification

The prokaryotic community in the Lake Pavin sediment was dominated by *Bacteria*, and *Archaea* accounted for 3 to 12% of the total prokaryotic 16Sr DNA copy number (Figure 1D). According to the average 16S rDNA copy number in archaeal and bacterial genomes [42], *Archaea* accounted for 5 to 18% of the total prokaryotic cells. No significant trend was noted for archaeal abundance according to depth (Figures 1D and 3). Abundance of the dominant archaeal lineages detected in clone libraries, the euryarchaeotal lineages *Methanosaetaceae*, *Methanomicrobiales*, MBG-D and of the *Crenarchaeota,* was determined through the quantification of their 16S rDNA by qPCR (Figures 3 and 4B to D). The acetotrophic (*Methanosaetaceae*) and hydrogenotrophic (*Methanomicrobiales*) methanogens dominated the archaeal community in the uppermost layer (4×10^7^ and 1×10^7^ copies g^−1^ dry sediment at 0–2 cm depth, respectively) and decreased with depth (3.7×10^6^ and 4.7×10^6^ copies g^−1^ dry sediment at 36–38 cm, respectively, Figure 4B). Consistent with cloning and TTGE analyses, qPCR results showed a clear difference in archaeal community structure along the sediment core. The methanogenic populations were replaced by the MBG-D (2.6×10^7^ copies g^−1^ dry sediment at 36–38 cm depth, Figure 4D) and the *Crenarchaeota* (1.2×10^7^ copies g^−1^ dry sediment at 36–38 cm depth, Figure 4D). A strong correlation was noted between the abundances of these two lineages (r = 0.901, p<0.001, Table S2). The abundances of *Methanosaetaceae* and MBG-D groups were strongly negatively correlated (r = −0.835, p<0.001, Table S2) and exhibited a positive and slightly negative correlation with OM and OC content of the sediment, respectively (Table S2).

As RNA content is generally correlated to cell activity [51], the 16S rRNA of dominant archaeal lineages (*Methanosaetaceae*, *Methanomicrobiales*, MBG-D lineages and *Crenarchaeota*) were quantified and compared to their number of 16S rDNA in order to estimate how their activity changed with depth (Figure 7). The trends were consistent with those of 16S rDNA quantification for *Methanosaetaceae*, *Methanomicrobiales* and MBG-D but not for *Crenarchaeota* (Figures 4D and 7A). The ratios of 16S rRNA to 16S rDNA copy number were consistent with those of the 16S rRNA quantification (Figures 7A and B).

## Discussion

### *Archaea* are not the Dominant Component of the Prokaryotic Community in Freshwater Sediments

*Archaea* is most likely the dominant microbial domain of the deep marine subsurface [10], [52]. In lake sediments, archaea account from less than 1% [13] to 99% [53] of the prokaryotic community. According to available data, archaea were found to be dominant in sediments of saline lakes [e.g. Lake Chaka [54], Lake Qinghai [55]] and to be a minority component of the prokaryotic community in freshwater sediments [13], [56], [57]. Agreeing with these previous observations, our q-PCR analysis demonstrated that archaea accounted for 5–18% of the prokaryotic community throughout the 40-cm core of the freshwater sediments of Lake Pavin. Whereas few studies reported quantification of archaea in lake sediments, discrepancies in the relative proportion of archaea according to salinity might be explained, in part, by Valentine’s hypotheses [58]. In this interesting opinion paper, the author postulated that “*adaptation to chronic energy stress is the primary factor differentiating archaeal and bacterial ecology*”. In freshwater sediments, such as in Lake Pavin, where energetic stress is not “chronic” compared to saline lakes, the high degree of adaptability and metabolic diversification of bacteria would allow them to dominate archaea.

### *Archaea* Exhibited a Dichotomic Distribution

Whereas, no significant vertical variations in the abundance of the overall archaea were noted, important depth-related changes occurred in the composition and structure of the archaeal community. Such vertical changes in the structure of the archaeal community were reported from freshwater lake sediments according to fingerprint analyses [53], [59], but without clear determination of how each archaeal lineage is affected by depth in the sediment. In our study, the gradual shift of archaeal community composition along the Lake Pavin sediment core was supported by TTGE patterns, phylogenetic and quantification analyses.

Changes in archaeal community composition were accompanied by an increase in both richness and diversity between the upper and the deeper layers of the sediment. Compared to available data, the archaeal richness in Lake Pavin sediments (35 phylotypes in average) was high (e.g. Lake Hovsgol-14 phylotypes [60]; Lake Biwa-10 phylotypes [59], Lake Taihu-19 phylotypes [53], Lake Kivu-12 phylotypes [61]). Deep sediment layers, although with binding conditions, supported a diverse archaeal community. In undisturbed ecosystems like deep lake sediments, inactive cells may accumulate and persist during a long period after growth, depending on their resistance to starvation [62]. In these cold anoxic environments, the majority of DNA is extracellular and may be preserved for thousands of years [63], [64]. However, two observations show that the high diversity of archaea in the deep layers was not artefactual: (1) a clear dichotomy in the distribution of archaeal populations with methanogenic archaea progressively replaced by other archaeal clades (putatively non-methanogenic), (2) 16S rRNA quantification revealed that a large proportion of targeted communities are seemingly active (with the exception of *Crenarchaeota*).

All of these observations (diversity, composition, and activity patterns of archaeal communities along the sediment core) suggest that more ecological niches, clearly different from those in the surficial sediment, are available for archaeal taxa in the deep sediment layers. Notably, the clear inverse trend between methanogenic and uncultured archaeal lineage (putatively non-methanogenic) abundances with depth suggests they have contrasted ecological niches within the sediment. Several studies on archaeal communities inhabiting lake sediments also reported an inverse correlation between the relative proportions of *Methanosaeta* and *Methanomicrobiaceae*, and those of MBG-D [9], [12], [13], [55], [57], [59], [61], [65].

### Which Archaeal Groups Occurred and What can they do?

As outlined above, methanogenic archaea were progressively replaced along the sediment core by archaeal clades with unknown metabolisms. It is exciting as well as frustrating to detect so many archaeal clades for which no information about their metabolic function currently exists.

#### Methanogenic archaea

According to our data, it is clear that the main archaeal metabolic function in the surface sediment layer (0–4 cm) of Lake Pavin is methane production. Methanogen dominance in this layer is consistent with Lake Pavin meromicticity, as the reduction of the inorganic electron acceptors (i.e. sulfate, ferric iron, nitrate…) occurs in the anoxic water column [17]. According to the high methane concentration in the sediment (Figure 1B) and to the high abundance and activity of the methanogens in the superficial sediment layer, where mineralization is the most intensive, methanogenesis is expected to be the most important terminal mineralization pathway in this ecosystem. This terminal-electron-accepting pathway accounts, in freshwater system, for 10 to 50% of organic matter mineralization [66].

In Lake Pavin sediments, methanogenesis is performed by two known methanogenic lineages: the acetotrophic *Methanosaetaceae* and the putatively hydrogenotrophic *Methanomicrobiales*. These lineages are frequently observed in superficial zone of freshwater sediments [13], [53], [57], [59], [67]. The genus *Methanosaeta* and the family *Methanoregulaceae* have been also found to dominate freshwater sediments [68]. The abundance of *Methanosaetaceae* was significantly correlated with both OM and OC content along the sediment core (Table S2). In contrast with the abundance of *Methanosaetaceae*, the content in OM and OC did not decrease significantly with depth (Figure 1C). This suggests that another factor, probably the organic matter lability, is responsible for the decrease in the abundance of *Methanosaetaceae*. This hypothesis is supported by the decrease of the 16S rRNA to 16S rDNA ratio (Figure 7B). Methanosaeta members are only able to grow on acetate, and considering that the organic matter reactivity decreases with sediment age and depth [69], and that dissolved labile compounds only diffuse on a limited surface layer [70], cells detected in the deepest layers are probably not growing but rather surviving. The abundance of *Methanomicrobiales* is less negatively correlated to depth than that of *Methanosaetaceae* (Table S2). Chan *et al*. [57] hypothesized that this differential response of *Methanomicrobiales* and *Methanosaetaceae* to increasing depth may be due to a differential death (starvation) rather than a differential growth.

Another surprising observation is the detection of high methane concentrations in the deep layer of the sediment core where known methanogenic archaea were detected at low relative abundances and proportions. We cannot exclude that methane detected in the deep layer is an older CH_4_ that is physically entrapped but, the methane profile, with concentrations decreasing in the intermediate layer (Figure 1B), suggests that archaeal groups other than known methanogenic lineages might be involved in the methane cycle. This hypothesis is discussed further in the sections below.

#### Uncultured archaeal clades

Uncultured archaeal lineages are ubiquitous in freshwater sediments (Table S3, [61], [71], [72]) suggesting that they are adapted to this type of environment where they might play key functional roles. In Lake Pavin, the relative proportion and abundance of MBG-D and MCG increased with depth and these lineages dominated the archaeal community in the deeper layers of the sediment. A similar trend was also reported, for MBG-D, according to clone libraries from marine [73], freshwater [9] and hypersaline [65] sediments, and from a peatland [74]. According to their environmental distribution, mainly in sub-surfaces (Table S3), these archaeal lineages are probably able to use intriguing metabolic pathways and probably have special physiological adaptations allowing them to thrive with the low energetic flux available in buried sediment layers. But, these adaptations might reduce the competiveness of these archaea in dynamic and energetic environments as they are less abundant in surface sediment layer where labile organic matter is seasonally delivered to the sediment by dead algal deposition.

Sequences belonging to the euryarchaeotal the Marine benthic Group-D, a Thermoplasmatales-related group, were found in a variety of freshwater and marine habitats [9], [74]–[76]. Along with Rice Cluster V (RC-V) and Lake Dagow Sediment (LDS) lineages [72], MBG-D is the most widely encountered uncultured lineage in freshwater lake sediments (Table S3). Although the metabolism of MBG-D representatives remains unknown, several hypotheses may be postulated according to their environmental repartition and their phylogenetic affiliation. The high methane concentration in the deeper layer might be consistent with a methanogenic activity of MBG-D, although this group was previously identified as non-methanogenic [55]. MBG-D represented the unique archaeal members in a clone library from the bottom of the AOM zone of the Lake Cadagno [9] and were detected in several other marine environments and cultures where AOM occurs [76]–[78]. Beal *et al*., [79] also noted that MBG-D clones represented up to 40% of archaea in a clone library of their AOM enrichments from methane-seep sediment. These observations might imply the involvement of MBG-D members in AOM. This hypothesis is consistent with the high activities of MBG-D in the intermediate layer of Pavin sediments where methane concentrations decreased (Figure 1B and 7). However MBG-D also represents a high fraction of the prokaryotic community in hypersaline sediments where methane concentrations are extremely low [55], [65], suggesting that some MBG-D members are not involved in AOM processes but instead, would benefit from waste-products, intermediates or dead cells produced in sedimentary environments. As proposed for members of two lineages related to MBG-D, the *Thermoplasmatales* [80] and RC-III (a sister group of MBG-D, [81]), representatives of MBG-D might be scavengers in their environment and may use components from decaying microorganisms (such as oligopeptides [82]) as carbon and/or energetic sources for growth. For example, the decay of methanogenic microorganisms with depth might provide building elements for some representatives of uncultured archaeal lineages (as isoprenoids moieties of lipids) that may minimize their energy expenditure for growth and maintenance [83].

The Miscellaneous Crenarchaeal Group is a cosmopolitan group, frequently retrieved in anoxic habitats [10], [84] and have been considered as heterotrophic anaerobes based on their capability to take up organic carbon in buried sediments [84]. However, their huge cosmopolitan distribution in a wide range of biogeochemically distinct sedimentary settings [84] and their complex phylogeny suggest a metabolic diversity and an ecosphysiological flexibility larger than assumed previously. Current evidence suggests that some members of the MCG lineage may obtain energy from the anaerobic oxidation of methane but use a “dissimilatory” methane-oxidizing process and do not assimilate its carbon [84]. This hypothesis fits with the decrease of methane concentrations in the intermediate layers of the sediment core of Lake Pavin where MCG dominated the archaeal community. However, the achievement of other metabolic pathways by MCG occurring in Lake Pavin sediments is not excluded.

### Conclusion

This study revealed that important changes in the archaeal structure occurred in the sediment of Lake Pavin along the 40 cm sediment core. The opposite trends between methanogens and uncultured lineage abundances may be explained by differing or opposite optimum growth conditions. It could also be hypothesized that the decay of methanogenic microorganisms with depth furnishes building elements for some representatives of uncultured archaeal lineages. One of these uncultured archaeal groups, MBG-D, is generally associated with marine and hypersaline environments, however, its high abundance in Lake Pavin sediments and its common occurrence in freshwater lake sediments demonstrate that it can be an important group in sedimentary environments regardless of the salinity. The common association of MBG-D with communities performing AOM, in a number of environments and/or culture enrichments, presents the intriguing hypothesis that AOM also occurs in Lake Pavin sediments, though this needs further study. The new primers designed herein and the probes recently designed [9] would be helpful to clarify the role MBG-D plays in this significant ecological process.

## Supporting Information

Figure S1 **Specificity and binding sites (shading) of primers 490f and 818r targeting MBG-D**. These features are shown on a partial alignment of representative archaeal 16S rDNA sequences from positions 490 to 508 and from 818 to 837 (*E. coli* numbering), respectively.
(DOC)

Figure S2 **Hierarchical cluster analysis and ANOSIM statistics on TTGE profiles.** (A) Hierarchical cluster analysis, performed from TTGE banding patterns for each sample of the sediment core 1 (Fig. 1A), using the Jaccard coefficient and the UPGMA method. The dashed vertical line indicates the distance that was chosen for cluster separation. (B) ANOSIM statistics for comparisons of communities using TTGE similarity values. Upper, Intermediate and Deeper refers to clusters defined in Figure 2.
(DOC)

Table S1 **Number and affiliation of archaeal 16S rRNA gene sequences identified in clone libraries.** Data were obtained on samples from sediment core 1. MBG-D: Marine Benthic Group D; RC-V: Rice Cluster V; DSEG: Deep Sediment Euryarchaeotal Group; Val-III: Valkea-III; MCG: Miscellaneous Crenarchaeotal Group; MGI: Marine Group I; MBG-B, -A: Marine Benthic Group B and A.
(DOC)

Table S2 **Spearman rank correlations between the abundance of prokaryotic groups, depth, OM and OC.** Only correlations with p value <0.05 are shown. *p value <0.01. **p value <0.001. OM, Organic matter content, OC, Organic carbon content, Mst: *Methanosaetaceae*, MM: *Methanomicrobiales*, Cre: *Crenarchaeota*, Arc: *Archaea*, Bac: *Bacteria*; MBG-D: Marine Benthic Group D.
(DOC)

Table S3 **Occurrence of the main uncultured euryarchaeotal and crenarchaeotal/thaumarchaeotal lineages in freshwater lake sediments.** 16S rRNA gene sequences used for this table were previously published. The affiliation was based on published trees and new construction of trees using archaeal sequences from lake sediments retrieved from NCBI. “×” : detected; “−” : undetected; “?” ambiguous affiliation. MBG-D: Marine Benthic Group D, LDS: Lake Dagow Sediments, RCIII: Rice Cluster III, RCV: Rice Cluster V, DSEG: Deep Sediment Euryarchaeotal Group, Val-III: Valkea-III, MCG: Miscellaneous Crenarchaeotal Group, MBG-A: Marine Benthic Group A, MBG-B: Marine Benthic Group B, MGI: Marine Group I.
(DOC)
