# Supplementary material for: Stratification of Archaea in the Deep Sediments of a Freshwater Meromictic Lake: Vertical Shift from Methanogenic to Uncultured Archaeal Lineages
Source: PLoS One. 2012 Aug 21;7(8):e43346. doi: 10.1371/journal.pone.0043346 (PMC3424224; doi:10.1371/journal.pone.0043346)

**Figure S.2. Specificity and binding site (shading) of primers 490f and 818r targeting MBG-D.** These features are shown on a partial alignment of representative archaeal 16S rDNA sequences from positions 490 to 508 and from 818 to 837 (*E. coli* numbering), respectively.


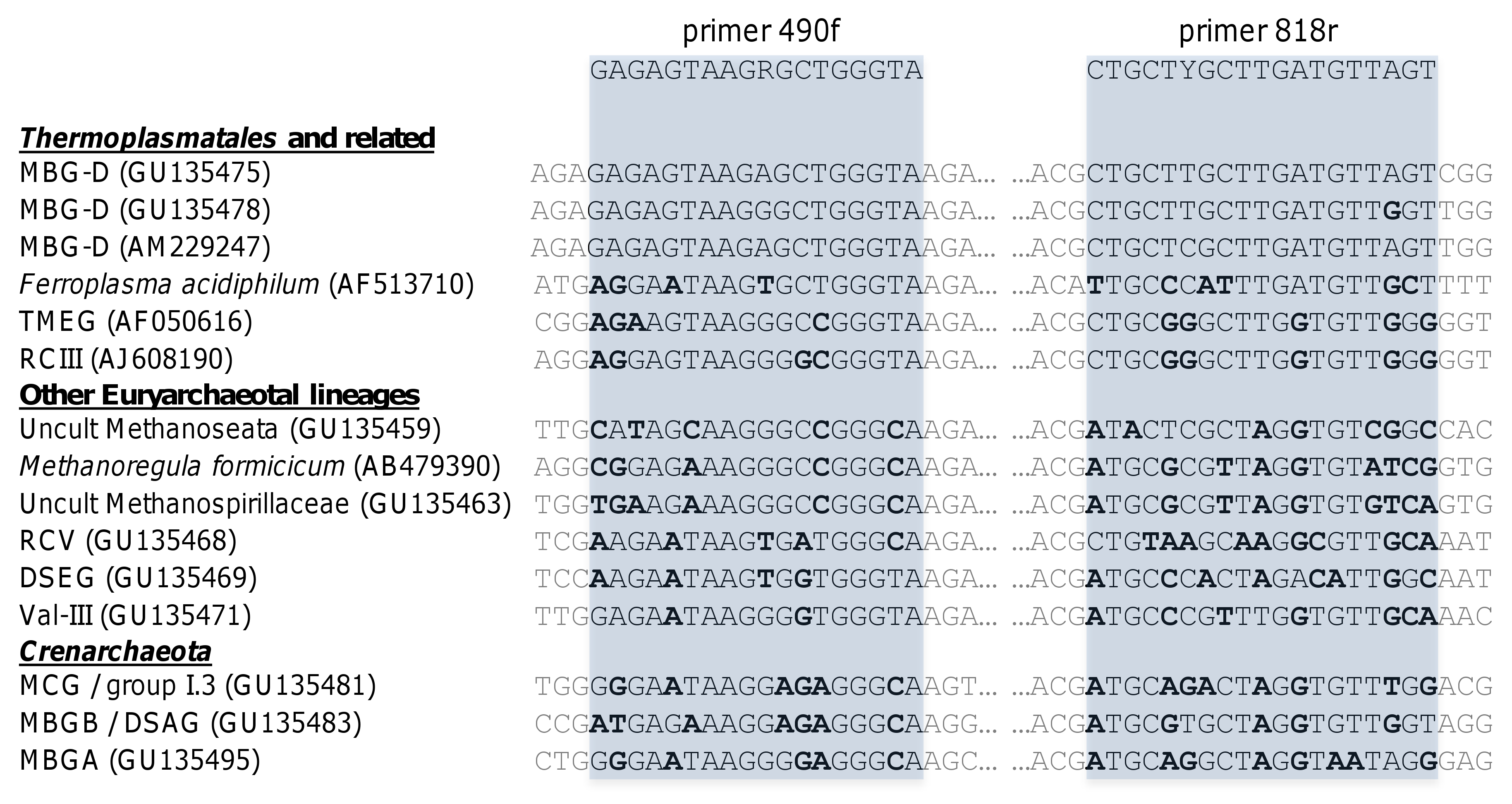

Supplement: Figure S1 — Specificity and binding sites (shading) of primers 490f and 818r targeting MBG-D. These features are shown on a partial alignment of representative archaeal 16S rDNA sequences from positions 490 to 508 and from 818 to 837 (E. coli numbering), respectively. (DOC) [file pone.0043346.s001.doc]
