# Supplementary material for: Stratification of Archaea in the Deep Sediments of a Freshwater Meromictic Lake: Vertical Shift from Methanogenic to Uncultured Archaeal Lineages
Source: PLoS One. 2012 Aug 21;7(8):e43346. doi: 10.1371/journal.pone.0043346 (PMC3424224; doi:10.1371/journal.pone.0043346)
Supplement: Figure S2 — Hierarchical cluster analysis and ANOSIM statistics on TTGE profiles. (A) Hierarchical cluster analysis, performed from TTGE banding patterns for each sample of the sediment core 1 (Fig. 1A), using the Jaccard coefficient and the UPGMA method. The dashed vertical line indicates the distance that was chosen for cluster separation. (B) ANOSIM statistics for comparisons of communities using TTGE similarity values. Upper, Intermediate and Deeper refers to clusters defined in Figure 2. (DOC) [file pone.0043346.s002.doc]

**Figure S.2. Hierarchical cluster analysis and ANOSIM statistics on TTGE profiles.** (A) Hierarchical cluster analysis, performed from TTGE banding patterns for each sample of the sediment core 1 (Fig. 1A), using the Jaccard coefficient and the UPGMA method. The dashed vertical line indicates the distance that was chosen for cluster separation. (B) ANOSIM statistics for comparisons of communities using TTGE similarity values. Upper, Intermediate and Deeper refers to clusters defined in Figure 2.

B

|  | Upper *vs* intermediate | Upper *vs* Deeper | Intermediate *vs* deeper |
| --- | --- | --- | --- |
| **R** | 0.7863 | 0.8889 | 0.7227 |
| **P value** | 0.0051 | 0.096 | 0.0042 |
