# Supplementary material for: Stratification of Archaea in the Deep Sediments of a Freshwater Meromictic Lake: Vertical Shift from Methanogenic to Uncultured Archaeal Lineages
Source: PLoS One. 2012 Aug 21;7(8):e43346. doi: 10.1371/journal.pone.0043346 (PMC3424224; doi:10.1371/journal.pone.0043346)
Supplement: Table S1 — Number and affiliation of archaeal 16S rRNA gene sequences identified in clone libraries. Data were obtained on samples from sediment core 1. MBG-D: Marine Benthic Group D; RC-V: Rice Cluster V; DSEG: Deep Sediment Euryarchaeotal Group; Val-III: Valkea-III; MCG: Miscellaneous Crenarchaeotal Group; MGI: Marine Group I; MBG-B, -A: Marine Benthic Group B and A. (DOC) [file pone.0043346.s003.doc]

**Table S.1. Number and affiliation of archaeal 16S rRNA gene sequences identified in clone libraries from the sediment core collected in December 2007 in the Lake Pavin.**

| OTU name | Accession number | Number of clones | | | Phylogenetic affiliation | | |
| --- | --- | --- | --- | --- | --- | --- | --- |
|  |  | 0 -2 cm | 10 - 12 cm | 36 - 38 cm |  |  |  |
| Pav-sed-101 | GU135459 | 58 | 4 | 2 | *Methanosaetaceae* | *Methanosarcinales* | *Euryarchaeota* |
| Pav-sed-202 | GU135463 | 3 |  |  | *Methanospirillaceae* | *Methanomicrobiales* |  |
| Pav-sed-201 | GU135462 | 16 | 1 |  | *Methanoregulaceae* |  |  |
| Pav-sed-204 | GU135465 | 3 |  |  |  |  |  |
| Pav-sed-207 | GU135467 |  |  | 1 |  |  |  |
| Pav-sed-301 | GU135468 | 1 |  |  | RCV | |  |
| Pav-sed-302 | GU135469 |  | 1 |  | DSEG | |  |
| Pav-sed-304 | GU135471 |  | 1 |  | Val-III | |  |
| Pav-sed-401 | GU135475 |  | 9 | 28 | Thermoplasmatales-related | MBG-D |  |
| Pav-sed-402 | GU135476 |  | 2 |  |  |  |  |
| Pav-sed-403 | GU135477 |  | 8 | 1 |  |  |  |
| Pav-sed-404 | GU135478 |  | 1 |  |  |  |  |
| Pav-sed-501 | GU135481 | 4 | 6 | 2 | MCG | | *Crenarchaeota* |
| Pav-sed-506 | GU135486 |  | 7 | 5 |  |  |  |
| Pav-sed-508 | GU135488 |  | 4 | 1 |  |  |  |
| Pav-sed-509 | GU135489 |  | 3 |  |  |  |  |
| Pav-sed-511 | GU135491 |  | 1 |  |  |  |  |
| Pav-sed-514 | GU135494 |  | 3 |  |  |  |  |
| Pav-sed-516 | GU135496 |  | 1 |  |  |  |  |
| Pav-sed-517 | GU135497 |  | 2 |  |  |  |  |
| Pav-sed-518 | GU135498 |  |  | 2 |  |  |  |
| Pav-sed-521 | GU135501 |  |  | 1 |  |  |  |
| Pav-sed-522 | GU135502 |  |  | 1 |  |  |  |
| Pav-sed-502 | GU135482 | 2 |  |  | unidentified Crenarchaeota | |  |
| Pav-sed-513 | GU135493 |  | 3 |  |  |  |  |
| Pav-sed-504 | GU135484 |  | 1 |  |  |  |  |
| Pav-sed-510 | GU135490 |  | 1 |  |  |  |  |
| Pav-sed-507 | GU135487 |  | 1 |  |  |  |  |
| Pav-sed-519 | GU135499 |  |  | 1 |  |  |  |
| Pav-sed-515 | GU135495 |  | 2 |  | MBGA | |  |
| Pav-sed-503 | GU135483 | 1 | 1 |  | MBGB / DSAG | |  |
| Pav-sed-505 | GU135485 |  | 1 | 2 | MG-I | | *Thaumarchaeota* |

MBG-D, Marine Benthic Group D; RC-V, Rice Cluster V; DSEG, Deep Sediment Euryarchaeotal Group; Val-III, from Valkea; MCG, Miscellaneous Crenarchaeotal Group; MGI, Marine Group I; MBG-B, -A, Marine Benthic Group B, A.
