# Supplementary material for: Stratification of Archaea in the Deep Sediments of a Freshwater Meromictic Lake: Vertical Shift from Methanogenic to Uncultured Archaeal Lineages
Source: PLoS One. 2012 Aug 21;7(8):e43346. doi: 10.1371/journal.pone.0043346 (PMC3424224; doi:10.1371/journal.pone.0043346)
Supplement: Table S2 — Spearman rank correlations between the abundance of prokaryotic groups, depth, OM and OC. Only correlations with p value <0.05 are shown. *p value <0.01. **p value <0.001. OM, Organic matter content, OC, Organic carbon content, Mst: Methanosaetaceae, MM: Methanomicrobiales, Cre: Crenarchaeota, Arc: Archaea, Bac: Bacteria; MBG-D: Marine Benthic Group D. (DOC) [file pone.0043346.s004.doc]

**Table S.**2. Spearman’s rank Correlation between the abundance of prokaryotic groups, depth, OM and OC.

|  | **Depth** | **Mst** | **MM** | **Cre** | **MBG-D** | **Arc** | **Bac** |
| --- | --- | --- | --- | --- | --- | --- | --- |
| Depth |  | -0.781** | -0.5* | 0.524* | 0.866** | - | -0.46 |
| OM | - | 0.725** | - | - | -0.415 | - | - |
| OC | - | 0.621** | - | - | -0.426 | - | - |
| Mst |  |  | - | -0.642 | -0.835** | - | - |
| MM |  |  |  | - | - | 0.626 | 0.802** |
| Cre |  |  |  |  | 0.901** | - | - |
| MBG-D |  |  |  |  |  | - | - |
| Arc |  |  |  |  |  |  | 0.621 |

Only correlations with p value < 0.05 are shown. * p value < 0.01. ** p value < 0.001. OM, Organic matter content, OC, Organic carbon content, Mst: *Methanosaetaceae*, MM: *Methanomicrobiales*, Cre: *Crenarchaeota*, Arc: *Archaea*, Bac: *Bacteria*; MBG-D: Marine Benthic Group D.
