# Supplementary material for: Stratification of Archaea in the Deep Sediments of a Freshwater Meromictic Lake: Vertical Shift from Methanogenic to Uncultured Archaeal Lineages
Source: PLoS One. 2012 Aug 21;7(8):e43346. doi: 10.1371/journal.pone.0043346 (PMC3424224; doi:10.1371/journal.pone.0043346)
Supplement: Table S3 — Occurrence of the main uncultured euryarchaeotal and crenarchaeotal/thaumarchaeotal lineages in freshwater lake sediments. 16S rRNA gene sequences used for this table were previously published. The affiliation was based on published trees and new construction of trees using archaeal sequences from lake sediments retrieved from NCBI. “×” : detected; “−” : undetected; “?” ambiguous affiliation. MBG-D: Marine Benthic Group D, LDS: Lake Dagow Sediments, RCIII: Rice Cluster III, RCV: Rice Cluster V, DSEG: Deep Sediment Euryarchaeotal Group, Val-III: Valkea-III, MCG: Miscellaneous Crenarchaeotal Group, MBG-A: Marine Benthic Group A, MBG-B: Marine Benthic Group B, MGI: Marine Group I. (DOC) [file pone.0043346.s005.doc]

**Table S.3. Occurrence of the main uncultured euryarchaeotal and crenarchaeotal/thaumarchaeotal lineages in freshwater lake sediments based on 16S rRNA gene sequences available in the literature.**

| **Lake sediment** | **Reference** | **Uncultured euryarchaeotal lineages** | | | | | | **Uncultured crenarchaeotal/thaumarchaeotal lineages** | | | |
| --- | --- | --- | --- | --- | --- | --- | --- | --- | --- | --- | --- |
|  |  | RC-V | MBG-D | LDS | TMEG | RC-II | Others | MCG | MGI | MBG-B | Others |
| **Michigan** | MacGregor et al. (1997) Appl Environ Microbiol 63: 1178–1181. | - | - | - | - | - | - | - | X | - | - |
| **Rotsee** | Falz et al. (1999) Appl Environ Microbiol 65: 2402–2408. | X | - | - | - | X | - | - | - | - | - |
| **Kinneret** | Nusslein et al. (2001) Environ Microbiol 3: 460–470. | - | X | - | ? | - | - | - | - | - | - |
| **Heywood** | Purdy et al. (2003) Appl Environ Microbiol 69:3181–3191. | - | - | - | - | - | - | - | - | - | - |
| **Biwa** | Koizumi et al. (2004) FEMS Microbiol Ecol 48:285–292. | X | - | ? | - | - | - | - | X | - | - |
| **Dagow** | Chan et al. (2005) Environ Microbiol 7:1139–1149. | X | X | X | X | - | unclassif | - | - | - | - |
| **Kinneret** | Schwartz et al. (2007) Syst Appl Microbiol 30:239–254. | X | - | - | - | - | - | - | - | - | - |
| **Stechlin** | Conrad et al. (2007) Limnol Oceanogr 52:1393-1406. | X | X | X | - | - | - | X | - | - | - |
| **Taihu** | Ye et al. (2009) FEMS Microbiol Ecol 70:263–276. | X | X | X | X | - | SM-I | - | - | - | MCG related |
| **Mussara** | Conrad et al. (2010) Limnol Oceanogr 55:689–702. | - | X | X | - | X | - | X | - | - | unclassif |
| **Batata** |  | X | X | X | - | X | - | X | - | - | unclassif |
| **Geneva** | Haller et al. (2011) Water Res 45:1213–1228. | X | - | X | - | - | - | - | - | - | - |
| **Cadagno** | Schubert et al. (2011) FEMS Microbiol Ecol 76:26–38. | - | X | - | X | - | AAA | - | - | X | MBG-C |
| **Pavin** | This study | X | X | - | - | - | DSEG, Val-III | X | X | X | MBG-A |

The affiliation was based on published trees and new construction of trees with archaeal sequences from lake sediments retrieved from NCBI. "x" : detected; "-" : undetected; "?" ambiguous affiliation. MBG-D: Marine Benthic Group D, LDS: Lake Dagow Sediments, RCIII: Rice Cluster III, RCV: Rice Cluster V, DSEG: Deep Sediment Euryarchaeotal Group, Val-III: Valkea-III, MCG: Miscellaneous Crenarchaeotal Group, MBG-A: Marine Benthic Group A, MBG-B: Marine Benthic Group B, MGI: Marine Group I.
